# Youngest case of third ventricular anaplastic neurocytoma

**DOI:** 10.4103/0971-5851.71660

**Published:** 2010

**Authors:** Chinnikatti Shravan Kumar, D. N. Sharma, Kuldeep Sharma, K. P. Haresh, G. K. Rath

**Affiliations:** *Department of Radiation Oncology, Institute Rotary Cancer Hospital, All India Institute of Medical Sciences, New Delhi, India*

**Keywords:** *Anaplastic neurocytoma*, *MIB-1 labeling index*, *radiotherapy*, *synaptophysin*, *temozolamide*

## Abstract

A 6-year-old child presented to us with on and off headache and vomiting for 4 months. On examination, there was bilateral papilledema with mild intracranial hypertension but with no neurological deficits. Magnetic resonance imaging (MRI) showed third ventricular mass with obstructive hydrocephalus with possibility of glioma. The patient underwent gross tumor excision and histopathology confirmed anaplastic neurocytoma. The postoperative MRI showed residual disease. The patient treated with adjuvant radiotherapy and temozolamide chemotherapy.

## INTRODUCTION

Central neurocytoma is most commonly found in the anterior portion of the lateral ventricle (50%), followed by a combined presentation in the lateral and third ventricles (15%) or biventricular location (13%). Only 3% of the cases occur in the third ventricle alone. Childhood atypical neurocytomas are extremely rare tumors in the central nervous system. Since this entity was introduced in 1982 by the WHO, neurocytomas are also described as a rare intraventricular benign neuronal tumour of the brain. Neurocytomas constitute nearly one-half of the supratentorial intraventricular tumors in adults, but that amounts to <1% of all tumors of the central nervous system and its coverings.[1,2]

Neurocytomas that are supratentorial are often calcified brain tumors affecting young adults, and are typically located in the lateral ventricles in the region of the foramen of Monro. Clinically, the tumor causes signs of increased intracranial pressure, visual, mental disturbances and, occasionally, pyramidal or endocrine signs and symptoms. Rarely, neurocytomas may be discovered accidentally with no clinical symptoms.

A majority of the neurocytomas are benign. Approximately 25% of these rare central nervous system tumors are more aggressive, with an MIB-1 labelling index >2% or atypical histological features.[3,4]

Therapeutic options in the treatment of central neurocytoma are complete resection (CR), complete resection plus radiotherapy, incomplete resection (IR) and IR plus radiotherapy. The data suggest that CR leads to significantly better local control and survival than IR. After IR, patients benefit from postoperative radiotherapy and chemotherapy.[2]

Neurocytomas atypical either by their unusual topographical or histological presentation or by their poor prognosis has been frequently entitled in this way on synaptophysin positivity. Here, we present a rare case of atypical/anaplastic neurocytoma in the youngest patient in the literature.

## CASE REPORT

**Figure 1:**
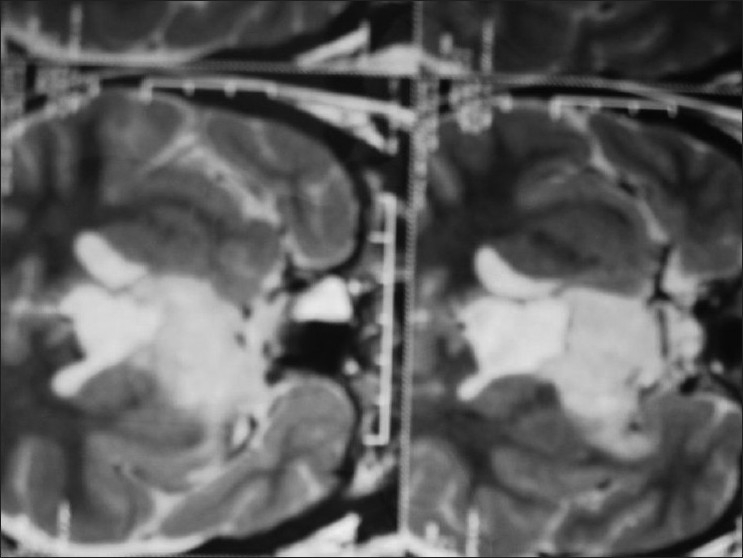
Pre-operative magnetic resonance imaging showing a mass in the third ventricle (3.5 cm × 3 cm × 2.7 cm)

We hereby report a 6-year-old young child who presented with on and off headache and vomiting of 4 months duration. There was no history of trauma, convulsions or loss/diminution of vision. On examination, there was presence of bilateral papilledema with mild intracranial hypertension and without neurological deficits. Preoperative magnetic resonance imaging (MRI) [Figure 1] showed a mass in the third ventricle (3.5 cm×3 cm×2.7 cm) extending into the body and frontal horn of the lateral ventricle, causing obstructive hydrocephalus with suspicion of possible gliomas. The patient underwent gross tumor excision and preoperative diagnosis of glioma.

Histopathology was confirmed to be anaplastic neurocytoma with synaptophysin positivity, and MIB-I labelling index is approximately 6% in the highest proliferating area, including vascular endothelial proliferation, increased proliferation index, mitosis and necrosis.

**Figure 2:**
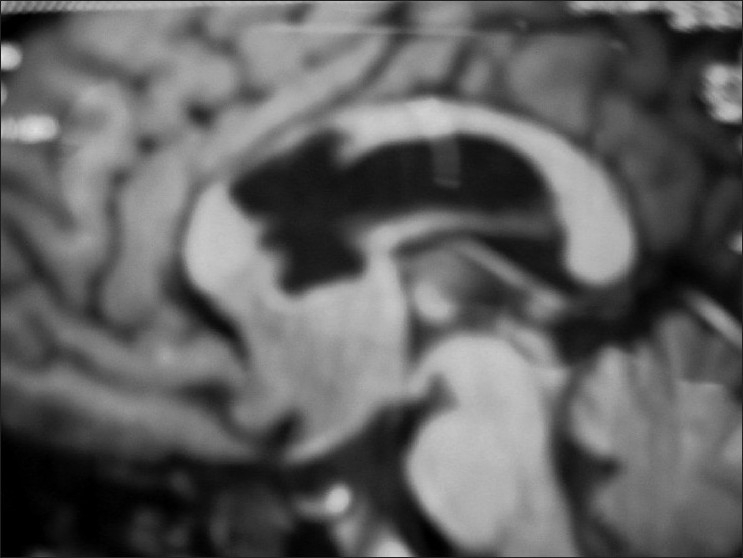
Post-operative magnetic resonance imaging showing the site and residual disease in the third ventricle

Postoperative MRI [Figure 2] showed residual lesion and then the patient received 60 gray in 30 fractions using advanced 3DCRT without concurrent temozolamide in view of the anticipitated increased toxicity in the young patient. The patient was followed-up for 6 weeks after the last fraction of radiation with contrast-enhanced MRI and MRI spectroscopy. There was no residual disease. This is further confirmed by GHA-SPECT, which reaffirms the fact that postoperative radiotherapy is an important aspect in complete eradication of residual disease after incomplete surgical removal of anaplastic neurocytoma.

## DISCUSSION

Until now, the reported literature from all over the world about atypical neurocytomas is around 90 cases in both children and adults. Most neurocytomas are well differentiated, being associated with better long-term survival than the more aggressive atypical lesions. Atypical neurocytomas are characterized by an MIB-1 labelling index >3% or atypical histological features.

As seen in the literature, the prognosis is worse for patients who have these atypical tumors compared with patients who have typical central neurocytomas. Thus, treatment should be more aggressive and should include both radiotherapy and chemotherapy apart from complete or near-CR.[5]

A review by Dirk Rades, Fabian Fehlauer, Steven E. Schild *et al*.[2] found that local failure occurred in 40% and tumor-related deaths were observed in 20% of the cases. The Kaplan-Meier curves demonstrated that CR, complete resection and radiotherapy (CR-RT) and incomplete resection and radiotherapy (IR-RT) were associated with better local control and survival rates compared with IR alone. The 3-year and 5-year local control rates were 73% and 57% after CR, 81% and 53% after CR-RT, 85% and 70% after IR-RT and 21% and 7% after IR, respectively.

Another study by Sharma *et al*.[6] in 20 cases of neurocytoma was selected over a 16-year period (1980-1995), which accounted for 0.28% of all intracranial tumors reported at his center. Treatment consisted of surgical resection (total 14, subtotal 6) followed by radiotherapy, demonstrating that there was no significant difference in any of these parameter values between histological groups I (benign histological characteristics) and II (showed mitoses + necrosis), except that the MIB-1 labelling index tended to be higher in group II tumors. Further, there was no significant correlation between these proliferative indices and the mitotic rate of the tumors as well as the survival of the patients. A longer follow-up will be required to determine the relationship between proliferative markers and outcome as well as to bring out any heterogeneity in their biological behavior.

A biopsy series of 36 cases by Soylemezoglu *et al*.[7] compared the clinical outcome. The mean size of the growth fraction, as determined by MIB-1 labelling index (MIB-1 LI) at first biopsy, showed a highly significant difference in the disease-free survival between the two groups (*P*=0.0068). Over an observation time of 150 months, there was a 22% relapse among patients with an MIB-1 LI less than 2% and a 63% chance of relapse among those with an MIB-1 LI greater than 2%, and they proposed the term “atypical central neurocytoma” for the latter subset, corresponding to WHO grade II.

A retrospective review by Mackenzie[8] identified 15 cases of central neurocytoma. Clinical follow-up was available for 14 patients. The proliferation potential of central neurocytoma is a useful predictor of clinical outcome, whereas histological atypia alone is not prognostically significant. It would be appropriate to recognize a subgroup of central neurocytomas with elevated proliferation potential. The terms “atypical” and “anaplastic” are not appropriate to describe these lesions as they imply a certain histologic appearance. The most accurate designation would be “proliferating neurocytoma.”

Ashkan *et al*.[9] presented prospective data on 12 patients with tumors diagnosed as central neurocytoma to highlight the diverse nature of this tumor and to challenge the classic notion in terms of clinical, radiologic and histologic data collected, and the Karnofsky performance score was evaluated for each patient. It was suggested that the traditional concept of central neurocytoma as a benign intraventricular tumor warrants reconsideration.

Brandes *et al*.[10] used chemotherapy regimen with etoposide 40 mg/m^2^/day for 4 days, cisplatin 25 mg/m^2^/day for 4 days and cyclophosphamide 1,000 mg/m^2^ on day 4. This cycle was repeated every 4 weeks. Stabilization of disease was observed in two patients and complete remission was observed in one patient. At last follow-up, these responses had been maintained for 15 months, 18 months and 36 months, respectively.

To our knowledge, only very little experience has been published to date concerning chemotherapy in the management of patients with atypical central neurocytomas. Thus, the value of chemotherapy remains to be defined.

In the above-reported case, we extrapolated the use of temozolamide (TMZ) in anaplastic astrocytomas. However, we could not use temozolamide (TMZ) in a concurrent setting as well as in an adjuvant setting with anticipation of increased toxicity to the growing brain and thereby avoiding delayed side-effects.

## CONCLUSION

Atypical (third ventricular) location, early younger age and histopathological features are some of the unusual, interesting findings in this case, which need to be published for further reference. And, this unique case also reestablishes the role of postoperative radiotherapy in incompletely resected anaplastic neurocytomas. To the best of our knowledge, this is the youngest patient of third ventricular anaplastic neurocytoma available in the literature.
